# Long non‐coding RNA NEAT1 transported by extracellular vesicles contributes to breast cancer development by sponging microRNA-141-3p and regulating KLF12

**DOI:** 10.1186/s13578-021-00556-x

**Published:** 2021-04-05

**Authors:** DaoPing Zhou, Juan Gu, YuePing Wang, HuaiGuo Wu, Wei Cheng, QingPing Wang, GuoPei Zheng, XueDong Wang

**Affiliations:** 1 Center for Precision Medicine, Anhui No.2 Provincial People’s Hospital, Hefei, 230041 Anhui China; 2 Department of Medical Laboratory Science, The Fifth People’s Hospital of Wuxi, Nanjing Medical University, 1215 Guangrui Road, Wuxi, 214000 Jiangsu China; 3 Department of Pathology, The Fifth People’s Hospital of Wuxi, The Medical School of Jiangnan University, Wuxi, 214000 Jiangsu China; 4 Department of Biology, College of Arts & Science, Massachusetts University, Boston, 02125 MA USA

**Keywords:** Breast cancer, Extracellular vesicles, Nuclear paraspeckle assembly transcript 1, microRNA-141-3p, Kruppel‐like factor 12, Invasion, Metastasis, Chemotherapy resistance

## Abstract

**Objective:**

Breast cancer (BC) remains a public-health issue on a global scale. Long non-coding RNAs (lncRNAs) play functional roles in BC. This study focuses on effects of NEAT1 on BC cell invasion, migration and chemotherapy resistance via microRNA (miR)-141-3p and KLF12.

**Methods:**

After extraction and identification of serum extracellular vesicles (EVs), NEAT1 expression in EVs was detected and its association with clinical characteristics of BC patients was analyzed. Besides, the gain-of function was performed to investigate the roles of NEAT1 and miR-141-3p in BC, and levels of NEAT1, miR-141-3p, KLF12 and MDR1 after EV treatment were detected by RT-qPCR and Western blot analysis. Furthermore, the in vitro findings were confirmed via lung metastases in nude mice.

**Results:**

NEAT1 expression in serum EVs was high and related to lymph node metastasis, progesterone receptor, estrogen receptor and Ki-67 in BC patients. After EV treatment, NEAT1 and KLF12 levels were increased, miR-141-3p expression was decreased, the abilities of proliferation, invasion, migration and in vivo metastasis were enhanced, and the sensitivity of cells to cisplatin, paclitaxel and 5-fluorouracil was decreased. After NEAT1 interference, NEAT1 and KLF12 levels in BC cells treated with EVs were decreased, miR-141-3p expression was increased, cell proliferation, invasion, migration and in vivo metastasis were decreased, and drug resistance sensitivity was increased. NEAT1 can bind to miR-141-3p and upregulates KLF12 expression.

**Conclusions:**

EVs inhibit the regulation of KLF12 by miR-141-3p by transporting NEAT1 to BC cells, thus promoting BC cell invasion, migration, and chemotherapy resistance.

## Introduction

Breast cancer (BC) is the main cause of cancer-related mortality among females over the world [1]. Based on morphological and molecular observation, BC is classified into at least five subtypes: luminal A, luminal B, HER2^+^, basal, and normal [2]. The symptoms of BC depend on the site of metastasis, of which depression, fatigue, insomnia, and pain are the frequent signs [3]. Kinds of risk factors are involved in the occurrence of BC, including overweight, alcohol consumption and physical inactivity, never having children, recent use of oral contraceptives and long menstrual history [4]. At present, targeted therapy, radiation therapy, chemotherapy, endocrine therapy, and surgery are the main treatments for BC [5]. However, BC patients are prone to suffer from tumor metastasis, drug resistance and infection [6]. Therefore, finding novel diagnosis and treatment of BC has become increasingly important.

Extracellular vesicles (EVs), bioactive tiny membrane bound vesicles released by cells’ endosomal compartment in tumor microenvironment [7], including exosomes and microvesicles, contain mRNA, microRNAs (miRs), lipids and proteins, have profound impacts on intercellular communication during drug resistance, tumor angiogenesis and metastasis [8–10]. Many clinical researches indicate that the tumor-derived EVs and their contents have great potential as a diagnostic tool for prostate cancer and BC [11, 12]. Increasing evidence indicates that cancer-secreted EVs play an important role in tumor metastasis and chemoresistance by transmission of long non-coding RNAs (lncRNAs) [13, 14]. The nuclear paraspeckle assembly transcript 1 (NEAT1), a newly discovered nuclear-restricted and cancer-associated lncRNA, has key functions in the development of several cancers [15], showing high expression in colorectal cancer, nasopharyngeal carcinoma and BC [16]. In this study, through bioinformatics prediction and dual-luciferase assay, we found that NEAT1 can sponge miR-141. miR-141, together with miR-200abc, miR-141 and miR-429, main members of miR-200 family, is downregulated in BC cells, and its inhibition increases mammary gland hyperplasia and tumorigenecity of BC cells [17]. In this study, dual-luciferase assay results indicated that Kruppel-like factors (KLF)12 is the target gene of miR-141. KLF, a class of zinc-finger transcriptional regulators [18], shows important roles in various cellular processes, such as cell growth, migration and cell cycle of various tumor types [19]. KLF12 has already been reported to repress gene expression by binding to gene promoter regions [20] and offer a potential diagnostic biomarker and therapeutic treatment for basal-like breast carcinoma [19]. Downregulation of KLF12 may suppress the growth of BC [21]. From all above, we speculate that there might be mechanisms of NEAT1 in serum EVs in invasion, migration and drug resistance of BC cells by regulating miR-141 and KLF12. Molecular and histochemical experiments were conducted in this study to testify the involvement of NEAT1 in serum EVs in cellular functions of BC, which may provide novel insights for therapeutic interventions in the future.

## Materials and methods

### Study subjects

From May 2017 to October 2018, 62 patients (28–68 years old, an average age of 49.4 ± 11.2 years old) with BC diagnosed and treated in Anhui No.2 Provincial People’s Hospital were enrolled in this experiment. Inclusion criteria were as follows: (1) all patients met the clinical diagnostic criteria for BC; (2) all patients were primary patients and never received radiotherapy or chemotherapy before operation; (3) all patients possessed complete clinical data. Patients complicated with other malignant tumors or organic diseases would be excluded. Thirty-eight women (12 cases of breast fibroma and 26 cases of breast diseases) (ranged from 20 to 65 years, average age: 46.2 ± 11.9 years old), with benign breast lesions diagnosed in the same period were enrolled, and they didn’t get any treatment prior to blood collection. Another 45 healthy women aged 25–70 years with an average age of 50.4 ± 11.0 years old showing no cardiovascular, liver, kidney, breast and other related diseases, were selected from the physical examination center of Anhui No.2 Provincial People’s Hospital during the same period. No significant differences were found in age among the three groups, which was comparable.

### Extraction and identification of serum EVs

A total of 5 mL fasting peripheral blood of each subject was collected. The supernatant was harvested after gradient centrifugation. The serum EVs were extracted in strict accordance with the TransEVs™ Serum/Plasma Exosome Kit (TransDetect, Beijing, China). Then protein concentration of EVs was determined using a protein DC assay kit (Thermo Fisher, Waltham, MA, USA) and the EVs were preserved at − 80 ℃. Protein concentration was used as the concentration standard of EVs.

Then EV morphology was observed under a transmission electron microscope (JEM-1200 EX,JEOL, Ltd. Tokyo, Japan). Afterwards, levels of EV surface protein markers CD9, CD63 and calnexin were determined by Western blot analysis, with the remaining supernatant after EV extraction as the negative control. The particle size and distribution of EVs were observed using nanoparticle tracking analysis (NTA). NEAT1 expression in EVs was detected by reverse transcription quantitative polymerase chain reaction (RT-qPCR), and its stability was tested. Additionally, RNAase was used to treat the EVs of each group to prove that NEAT1 was encapsulated in the EVs. SDS was used to treat the EVs and destroy the membrane structure to confirm the role of EVs.

### Cell culture and co‐uptake of EVs

Human BC cell lines MCF-7 and MDA-MB-231(the Cell Bank of Chinese Academy of Sciences, Shanghai, China), were incubated in Roswell Park Memorial Institute (RPMI)-1640 medium (Gibco, Grand Island, NY, USA) including 10% fetal bovine serum (FBS), penicillin (1 × 10^5^ U/L) and streptomycin (100 mg/L) at 37 ℃ with 5% CO_2_.

The EVs isolated from the serum of BC patients were incubated with PKH67 dyeing at 2–8 ℃ for 15–30 min. The supernatant was harvested after centrifugation, and samples were washed in PBS and resuspended. The suspension of 500 µL was detected on the flow cytometer at 490/502 nm. Then the stained EVs of 20 µL were respectively added into MCF-7 and MDA-MB-231 cells with 80% confluence. The entry of EVs into MCF-7 and MDA-MB-231 cells was observed under the confocal laser microscope (Olympus, Tokyo, Japan).

### Cell transfection

The EVs (0, 20, 40, 60 µg/mL protein concentration) were cultured in MCF-7 and MDA-MB-231 cells. The effects of EVs on MCF-7 and MDA-MB-231 cell proliferation were detected by 3-(4, 5-dimethylthiazol-2-yl)-2, 5-diphenyltetrazolium bromide (MTT) assay when culturing for 24, 48, and 72 h. MCF-7 and MDA-MB-231 cells were cultivated with 40 µg/mL EVs for 48 h. The drug resistance, invasion and migration of cells were detected with normal cultured cells as controls.

MCF-7 and MDA-MB-231 cells at logarithmic growth phase were transfected with negative control (NC) siRNA (si-NC), NEAT1 siRNA (si-NEAT1) (SS sequence: AGUUGUUAGUGUUGGUUAAGU, AS sequence: UUAACCAACACUAACAACUUA), mimic-NC and miR-141 mimic respectively, which were all constructed and synthesized by Sangon Biotech (Shanghai) Co., Ltd. (Shanghai, China). Transient transfection was conducted strictly based on the instructions of Lipofectamine 2000 (Invitrogen, Carlsbad, CA, USA). After 24 h, the expression was detected by RT-qPCR to verify the transfection effect.

### RT-qPCR

TRIzol (Invitrogen, Carlsbad, CA, USA) one-step method was utilized to extract total RNA in EVs and cells, and the high quality RNA was verified using ultraviolet analysis and formaldehyde denaturation electrophoresis. One µg RNA was reverse transcribed into cDNA by avian myeloblastosis virus reverse transcriptase. The qPCR was performed using SYBR Green method with glyceraldehyde-3-phosphate dehydrogenase (GAPDH) or U6 as an internal reference. The PCR primers were designed and synthesized by Sangon Biotech (Shanghai) Co., Ltd. (Shanghai, China) (Table 1). PCR system included cDNA 1.0 µL, 2 × SYBR Green Mix 10 µL, Forward Primer (10 µM) 0.5 µL, Reverse Primer (10 µM) 0.5 µL, and was supplemented into 20 µL with RNase free water. The reaction conditions were: pre-denaturation at 94 ℃ for 5 min, 40 cycles of denaturation at 94 ℃ for 40 s, annealing at 60 ℃ for 40 s, extension at 72 ℃ for 60 s, and finally extension at 72 ℃ for 10 min. The products were confirmed using agarose gel electrophoresis. The data were analyzed using 2^−ΔΔCt^ method. ΔΔCt = [Ct (target gene) − Ct (reference gene)] _experimental group_ − [Ct (target gene) − Ct (reference gene)] _control group_.

**Table 1:** Primer sequences of RT-qPCR

Genes	Sequences
NEAT1	F: 5’-TAACACGGTGCCTGGCTTAG-3’
	R: 5’-CTGTGTGTCAAAGCAAGGCC-3’
miR-141-3p	F: 5’-CGTCGCTAACACTGTCTGGTAA-3’
	R: 5’-GTGCAGGGTCCGAGGTATTC-3’
U6	F: 5’-GCTTCGGCAGCACATATACT-3’
	R: 5’-GGTGCAGGGTCCGAGGTATT-3’
KLF12	F: 5’-CTGCCTCCTCACCTTCTTCA-3’
	R: 5’-GGATGCGGTGAACATGACTC-3’
MDR1	F: 5’-TTGCTGCTTACATTCAGGTTTCA-3’
	R: 5’-AGCCTATCTCCTGTCGCATTA-3’
GAPDH	F: 5’-TGGGTGTGAACCATGAGAAG-3’
	R: 5’-GTGTCGCTGTTGAAGTCAGA-3’

*NEAT1 *nuclear paraspeckle assembly transcript 1, *miR *microRNA, *KLF12 *Kruppel-like factor 12, *MDR1 *multidrug resistance 1, *GAPDH *Glyceraldehyde-3-phosphate dehydrogenase, *RT-qPCR *reverse-transcription quantitative polymerase chain reaction

### Western blot analysis

The EVs/cells were lysed in radio-immunoprecipitation assay lysate and centrifuged with the supernatant obtained. The protein concentration was determined by the bicinchoninic acid protein quantitative detection kit (Wuhan Boster Biological Technology Co., Ltd, Wuhan, Hubei, China). The extracted proteins were supplemented with the loading buffer and placed in a water bath at 95 ℃ for 5 min. The proteins were separated using 10% sodium dodecyl sulfate (SDS) polyacrylamide gel electrophoresis (PAGE), and then transferred into the polyvinylidene fluoride (PVDF) membranes at 200 mA for 2 h. The membranes were sealed for 2 h, and probed with the primary antibodies CD9 (1:2000, ab223052), CD63 (1:1000, ab217345), calnexin (1:2000, ab10286, Abcam), KLF12 (1:5000, ab129459) and β-actin (1:5000, ab227387) (all from Abcam, Cambridge, MA, USA) at 4 ℃ overnight. Afterwards, the membranes were rinsed 3 times using Tris-buffered saline Tween 20 (TBST), each for 5 min, and supplemented with secondary antibody (ZSGB-Bio Co., Ltd, Beijing, China) for 1 h at room temperature. Subsequently, the membranes were washed 3 times, each for 5 min, and visualized with chemiluminescence reagent and Bio-Rad Gel Dol EZ imager (Bio-Rad Laboratories, Hercules, CA, USA). The target bands were analyzed with Image J software (National Institutes of Health, Bethesda, Maryland, USA) for grey value analysis.

### MTT assay

MCF-7 and MDA-MB-231 cell suspensions were diluted at an appropriate concentration and seeded at 5 × 10^4^ cells/well in 96-well plates, with 6 paralleled wells set in each group. When cell confluence reached 80%, the EVs (0, 20, 40, and 60 µg/mL) were added to MCF-7 and MDA-MB-231 cells. When culturing for 24, 48 and 72 h, cells were incubated with 20 µL 5 mg/mL MTT solution (Sigma, St. Louis, MO, USA) for 4 h at 37 ℃. Next, MTT solution was removed, and each well was added with 150 µL dimethyl sulfoxide (Sigma) and vibrated for 10 min. The optical density (OD) at 570 nm wavelength was tested using a microplate reader. The experiment was performed 3 times to get the average OD value.

MCF-7 cells were detached with 0.25% trypsin and centrifuged and counted. Later, cells were seeded at 5 × 10^4^ cells/well/100 mL in 96-well plates for 48 h with 40 µg/mL EVs. After that, cells were treated with different concentration of cisplatin (2, 4, 6, 8, 10 and 12 µmol/L), paclitaxel (0.5, 1.0, 1.5, 2.0, 2.5 and 3.0 µmol/L) and 5-fluorouracil (1, 2, 3, 4, 5 and 6 µg/mL) (Shandong Qilu Pharmaceutical Co., Ltd, Jinan, Shangdong, China) for 24 h later. Next, each well was added with 20 µL 5 mg/mL MTT solution and incubated at 37 ℃ for 4 h. The OD value was determined at 570 nm wavelength using a microplate reader. The inhibition rate of cell proliferation and 50% inhibitory concentration (IC50) were calculated, and the inhibition rate of cell proliferation (%) = [1 − (OD value of experimental group - OD value of control group)/(OD value of control group − OD value of blank well)] × 100%.

### Colony formation assay

MCF-7 cells were detached, suspended and counted. The cell suspension was diluted to the appropriate concentration and added to 6-well plates (2 mL/well). When cell clones grew to the appropriate size, the appropriate volume of drug was added and vibrated gently. Cells were cultured for 72 h continually and the culture media were removed. Subsequently, cells were washed 3 times (3 min/time) with pre-cooled PBS at 4 ℃ and added with methanol at 2 mL/well to fix cells for 15 min. After that, the methanol was absorbed and cells were gently washed with PBS 3 times (3 min/time), and put with 10% Giemsa dyeing diluted by PBS at 2 mL/well. Standing for 30 min at room temperature, the dyeing was sucked out, and cells were washed 3 times (3 min/time) with PBS again and dried naturally. Finally, the number of cell clones was counted under the microscope.

### Flow cytometry

MCF-7 cells were seeded at 1 × 10^5^ cells/well in 6-well plates. After 24 h of cell transfection or incubation with 40 µg/mL EVs, MCF-7 cells were cultured with appropriate volume of chemotherapeutic drugs for 48 h. After detachment, cells were centrifuged with the supernatant discarded, resuspended and washed in PBS, and the concentration was adjusted into 1 × 10^6^ cells/mL. A total of 200 µL cells were centrifuged after twice washes in 1 mL pre-cooled PBS. Then, cells were resuspended in 100 µL binding buffer, added with 2 µL Annexin-V- fluorescein isothiocyanate (FITC) (20 µg/mL), and mixed gently and placed on the ice for 15 min avoiding exposure to light. Afterwards, the cells were removed to the flow detection tube and added with 300 µL PBS. Each sample was added with 1 µL propidium iodide (PI) (50 µg/mL) before detection and tested on the flow cytometer within 30 min.

### Transwell assay

After diluting, 200 µL Matrigel (Becton, Dickinson and Company, Franklin lake, NJ, USA) was put into the apical chambers and air-dried at 4 ℃. MCF-7 cells were detached with 0.25% trypsin, and adjusted into 1 × 10^5^ cells/well using serum-free media. A total of 200 µL cell suspension was seeded into the apical chambers, and basolateral chambers were paved with the complete media containing 10% FBS. After 24-h cultivation at 37 ℃ in a 5% CO_2_ incubator, the Transwell chambers were removed and matrix and superficial cells in the apical chambers were erased with cotton swabs. After that, cells were fixed for 15 min with 4% polyformaldehyde and stained for 10 min with crystal violet. Five visual fields were taken and photographed randomly under the microscope, and the number of cells penetrating the membrane was counted. In the migration assay, the procedures were the same as above except that Matrigel was not put into the chambers.

### Scratch test

Behind the 6-well plates, uniform horizontal lines were drawn with a marker pen. About 5 × 10^5^ cells were put into each well, and cell confluence reached 100%. The next day, scratches were made with a 10 mL pipette tip vertical to the horizontal line behind the plates. After scratching, cells were cleaned with PBS gently to rinse and remove the scratched cells. Then cells were incubated with culture medium at 37 ℃ in a 5% CO_2_ incubator. Cells were collected at 0 h and 24 h, and photographed under the inverted microscope. The healing area of scratched wound was calculated using National Instrument Vision Assistant 8.6 software. Cell migration rate = the healing area of scratched wound/the initial scratched wound area × 100%.

### Lung metastases in nude mice

Six-week-old healthy specific pathogen-free (SPF) BALB/C nude mice (18 ± 2 g) were bought from Beijing Vital River Laboratory Animal Technology Co., Ltd. (Beijing, China) (SCXK (Beijing) 2016-0011). Well grown MDA-MB-231 cells were gathered, centrifuged, re-suspended with PBS, and adjusted to 1.0 × 10^6^ cells/mL. Each nude mouse was injected with 100 mL cell suspension via the tail vein (5 mice in each group) and fed in the SPF environment. From the first day of transplantion, nude mice in the EV group were given 50 µL of 1 mg/mL EVs via tail vein every 3 days, while nude mice in the control group were given 50 µL of PBS at the same time. On the 45th day, nude mice were executed by routine methods. The lungs were removed from the thoracic cavity, fixed in formaldehyde, embedded in paraffin, and made into sections at 5 µm. The size and number of lung metastases were observed using hematoxylin and eosin (HE) staining.

### Dual‐luciferase reporter gene assay

The binding sites of NEAT1 and miR-141-3p were predicted and analyzed by bioinformatics website, available at http://starbase.sysu.edu.cn/index.php [22]. NEAT1 3’UTR gene fragments were artificially synthesized and inserted into pMIR-reporter (Huayueyang Biotechnology Co., Ltd., Beijing China) using endonuclease sites Bamh1 and Ecor1. The mutation sites were designed based on NEAT1 wild type (WT). After restriction endonuclease digestion, the target fragments were inserted into pMIR-reporter plasmids using T4 DNA ligase. The correctly sequenced luciferase reporter plasmids WT and mutant (MUT) were co-transfected with mimic NC and miR-141-3p mimic to 293T cells (Shanghai Beinuo Biotechnology Co., Ltd, Shanghai, China) respectively using Lipofectamine 2000. Cells were gathered and lysed after 48-h transfection. Luciferase activity was determined using the luciferase detection kit (BioVision, San Francisco, CA, USA) and Glomax 20/20 luminometer fluorescence detector (Promega Corporation, Madison, WI, USA).

Bioinformatics software http://www.targetscan.org was applied to forecast the binding site between miR-141-3p and KLF12 3’UTR. KLF12 3’UTR sequence containing the binding site of miR-141-3p was synthesized to construct KLF12-WT. On the basis of KLF12-WT, binding sites were mutated to construct KLF12-MUT. KLF12-WT and KLF12-MUT plasmids were mixed with mimic NC or miR-141-3p mimic plasmids, respectively, and co-transfected into 293T cells. After transfection for 48 h, cells were gathered and lysed. Luciferase activity was determined using the luciferase detection kit.

### RNA-pull down assay

Biotin-labeled WT plasmid and MUT plasmid of miR-141-3p (50 nM each) were transfected into cells, respectively. After 48-h transfection, cells were gathered and washed with PBS, and then incubated with specific cell lysates (Ambion, Austin, Texas, USA) for 10 min. Residual lysates were incubated with M-280 streptavidin beads (Sigma) precoated with RNase-free and yeast tRNA (Sigma) for 3 h at 4 ℃. Then cells were rinsed with cold lysate twice, with low salt buffer three times, and once with high salt buffer. An antagonistic miR-141-3p probe was established as a control. Total RNA was extracted using TRIzol, and NEAT1 expression was determined by RT-qPCR.

### Statistical analysis

SPSS 21.0 (IBM Corp., Armonk, NY, USA) was applied for data analysis. Kolmogorov-Smirnov test showed whether the data were in normal distribution. The data are presented as mean ± standard deviation. Comparison between two groups was analyzed by *t* test, comparison among multiple groups was analyzed by one-way or two-way analysis of variance (ANOVA), and pairwise comparison after ANOVA was conducted by Tukey’s multiple comparisons test. Fisher’s exact test was utilized to compare the enumeration data. The *p* value was obtained using a two-tailed test and *p* < 0.05 meant a significant difference.

## Results

### Characterization of serum EVs

**Fig. 1:**
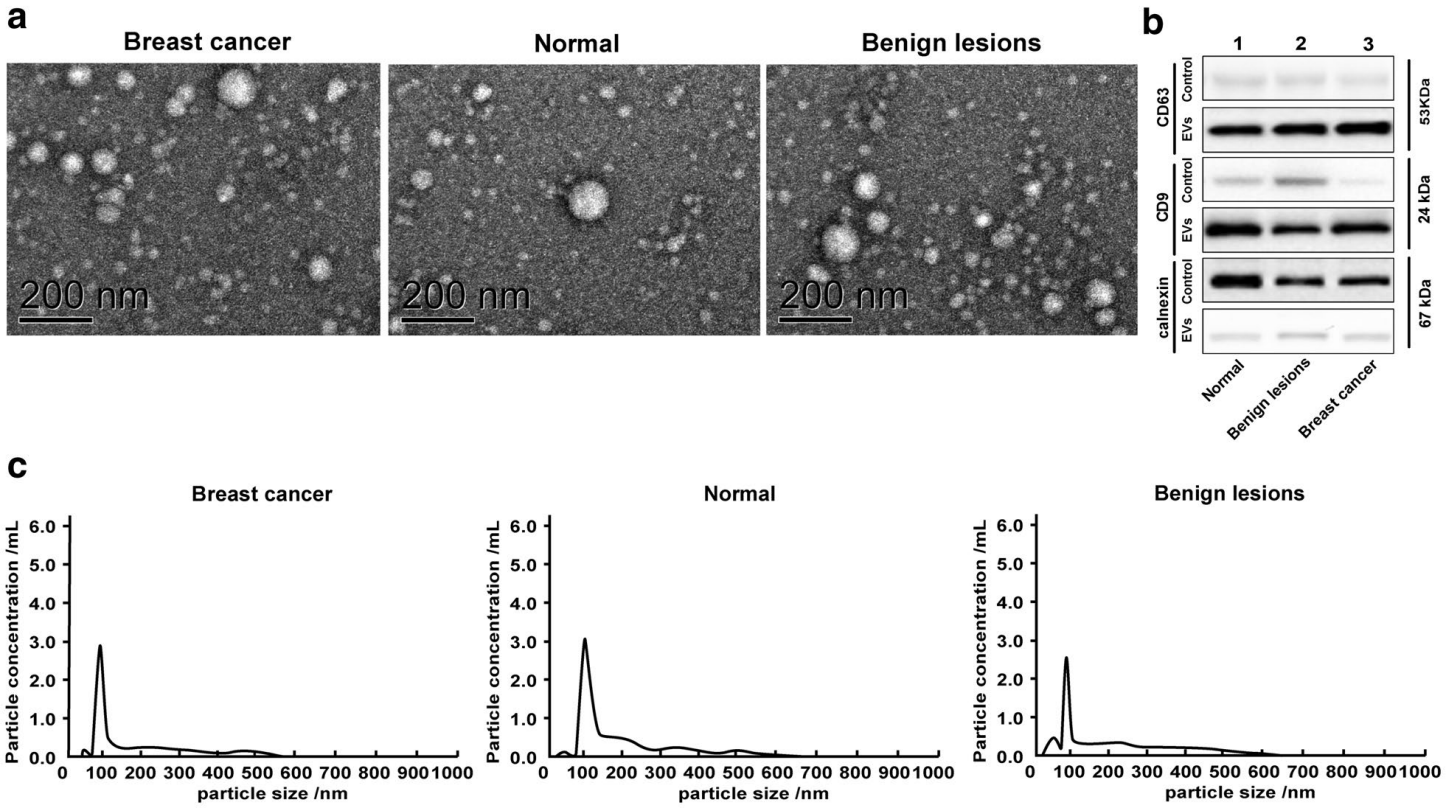
Identification of EVs. **a** The morphology of EVs was observed under the transmission electron microscopy; **b** Western blot analysis was utilized to detect levels of surface protein markers, CD9, CD63 and calnexin, in which 1 presented healthy control group, 2 presented benign individuals and 3 presented BC patients; the supernatant after EVs enrichment served as the control; **c** NTA was used to observe the size and concentration of EVs. Three independent experiments were performed

Typical round or oval cup-shaped EVs from different individuals with diameters ranging from 40 nm to 100 nm can be seen under the transmission electron microscopy, which were consistent with the size and shape of EVs reported in literatures (Fig. 1a).

Western blot analysis showed that EVs in serum of healthy controls, benign individuals and BC patients were rich in EV markers, and CD9, CD63 and calnexin were negative, indicating that the purity and abundance of serum EVs in this study were ideal (Fig. 1b).

NTA observed that the particle size of serum EVs from BC patients was 96.7 nm and the concentration of EVs was 2.9 × 10^10^/mL; the particle size of serum EVs from healthy controls was 101.6 nm and the concentration of EVs was 3.1 × 10^10^/mL; and the particle size of serum EVs from benign individuals was 95.1 nm and the concentration of EVs was 2.6 × 10^10^/mL; (Fig. 1c).

### High expression of NEAT1 in serum EVs of BC patients

**Fig. 2:**
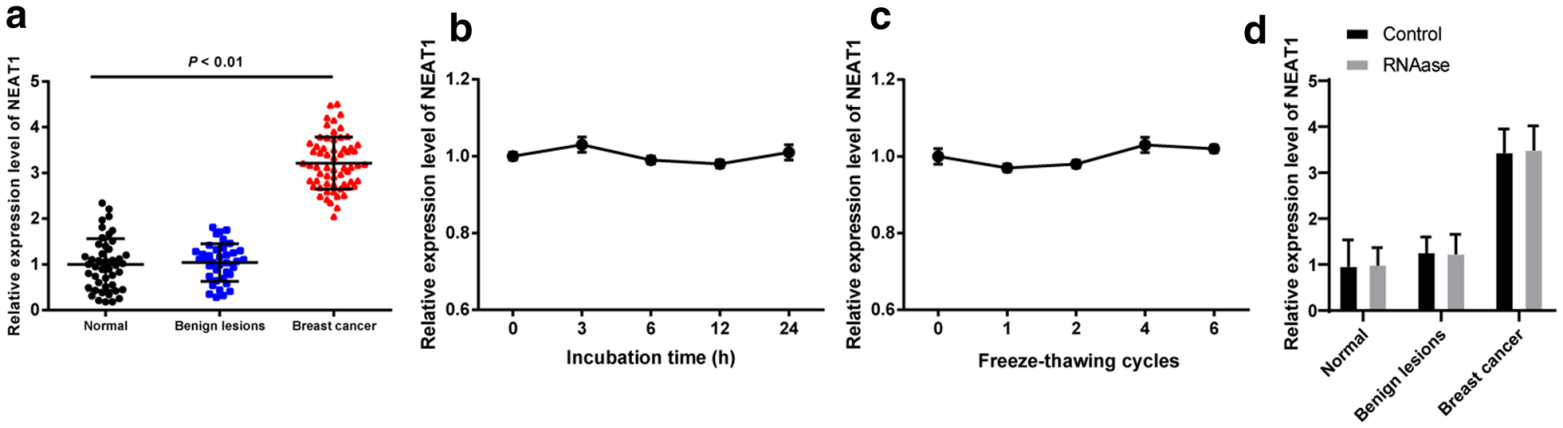
NEAT1 expression is higher in serum EVs of BC patient. **a** NEAT1 expression in serum EVs from healthy subjects, benign individuals and BC patients detected by RT-qPCR; **b** NEAT1 expression in serum EVs from BC patients at different time points under room temperature; **c** NEAT1 expression in serum EVs from BC patients at different freeze-thawing cycles; **d** NEAT1 expression in serum EVs from healthy subjects, benign individuals and BC patients before and after RNAase treatment. Three independent experiments were performed. One-way or two-way ANOVA was used for data analysis, and Tukey’s multiple comparisons test was used for pairwise comparisons after ANOVA

NEAT1 expression in serum EVs of BC patients was higher than that in benign individuals and healthy controls (all *p* < 0.05), but no significant difference was found in NEAT1 expression between benign individuals and healthy controls (*p* > 0.05) (Fig. 2a).

To investigate whether NEAT1 can stably exist in serum EVs of BC patients, the serum EVs of the same BC patients were divided into 5 fractions and placed at room temperature for 0, 3, 6, 12 and 24 h, respectively. No significant change was observed in NEAT1 expression in serum EVs at different time points (all *p* > 0.05) (Fig. 2b). The serum EVs of another BC patient were divided into 5 fractions, frozen at − 80 ℃, and thawed at room temperature for 0, 1, 2, 4 and 6 cycles, and the expression of NEAT1 in serum EVs did not change significantly after different freeze-thawing cycles (all *p* > 0.05) (Fig. 2c), indicating that NEAT1 can be relatively stable in the serum EVs of BC patients. Later, in order to prove that NEAT1 was contained in the EVs, the extracted EVs were treated with RNAase and then NEAT1 expression was detected using RT-qPCR. The results showed that NEAT1 expression had no significant change before and after RNAase treatment, indicating that NEAT1 expression was contained in the EVs (Fig. 2d).

Using the mean expression of NEAT1 as the critical value, BC patients were assigned into the high expression group (≥ 3.21) and the low expression group (< 3.21). The association between NEAT1 expression and clinical characteristics of BC patients was examined. The results showed that BC patients with high NEAT1 expression in serum EVs had higher lymph node metastasis rate, progesterone receptor (PR) positive rate, estrogen receptor (ER) positive rate and Ki-67 positive rate (all *p* < 0.05), but NEAT1 expression in serum EVs was not significantly related to the age, tumor size and clinical stage (all *p* > 0.05) (Table 2).

**Table 2:** The relationship between NEAT1 expression in serum EVs and clinical characteristics of BC patients [n (%)]

Clinical data	Cases	NEAT1 expression in serum EVs		*P*
		Low (n = 34)	High (n = 28)	
Age				0.317
< 50 years	29	18 (62.1)	11 (37.9)	
≥ 50 years	33	16 (48.5)	17 (51.5)	
Tumor diameter				0.127
≤ 2 cm	27	18 (66.7)	9 (33.3)	
> 2 cm	35	16 (45.7)	19 (54.3)	
Clinical stages				0.123
I + II	36	23 (63.9)	13 (36.1)	
III + IV	26	11 (42.3)	15 (57.7)	
Lymph node metastasis				0.03
Yes	41	18 (43.9)	23 (56.1)	
No	21	16 (76.2)	5 (23.8)	
PR expression				0.001
Positive	39	15 (38.5)	24 (61.5)	
Negative	23	19 (82.6)	4 (17.4)	
ER expression				0.010
Positive	35	14 (40.0)	21 (60.0)	
Negative	27	20 (74.1)	7 (25.9)	
Ki-67 expression				0.004
Positive	38	15 (39.5)	23 (60.5)	
Negative	24	19 (79.2)	5 (20.8)	

*NEAT1 *Nuclear paraspeckle assembly transcript 1, *BC *breast cancer, *EVs *extracellular vesicles, *PR *progesterone receptor, *ER *estrogen receptor

### High NEAT1 expression in EVs promotes BC cell invasion and migration

**Fig. 3:**
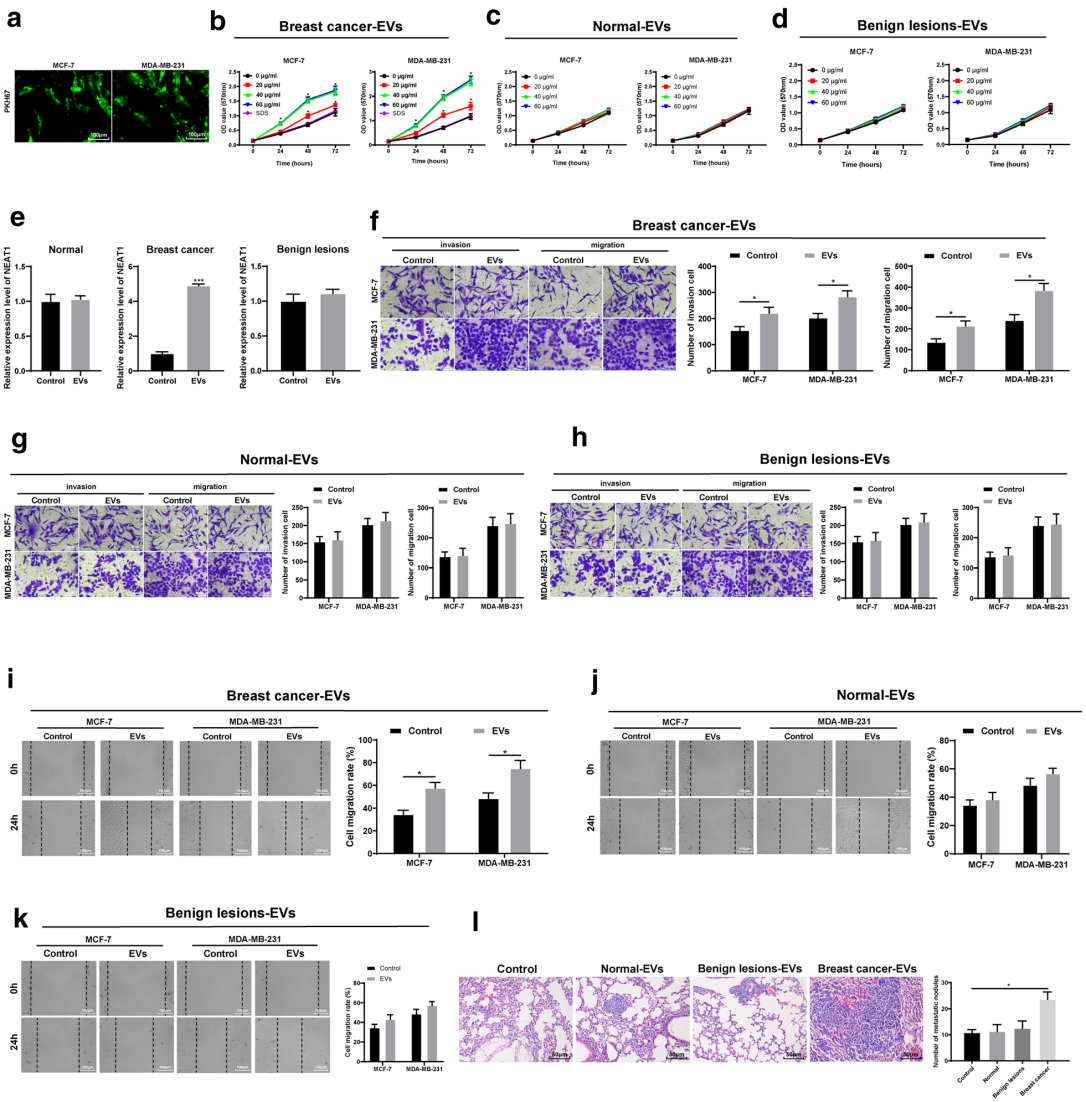
High NEAT1 expression in EVs promotes BC cell invasion and migration. **a** Laser scanning confocal microscopy was applied to observe the uptake of EVs by BC cells; **b**–**d**. MTT assay was applied to detect the effect of EVs on BC cell proliferation; **e** NEAT1 expression in MCF-7 cells before and after serum EV treatment detected by RT-qPCR; **f**–**h **Transwell assay was utilized to detect the effect of EVs on BC cell invasion; **i**–**k **Scratch test was used to detect effect of EVs on BC cell migration; **l **Lung metastases in nude mice were used to detect the effect of EVs on BC cell metastasis in vivo. n = 5; Three independent experiments were performed; control group is the remaining supernatant after EVs extraction; data in panel **e** were tested by *t* test, data in panels **b**, **c**, **d**, **f**, **g**, **h**, **i**, **j** and **k** were analyzed using two-way ANOVA, and data in panel **l** were analyzed using one-way ANOVA. Tukey’s multiple comparisons test was used for pairwise comparisons after ANOVA; * compared to the control group or 0 µg/mL, *p* < 0.05

Under the laser scanning confocal microscope, the serum EVs from BC patients with green fluorescent labeled by PKH67 were mainly seated in the cytoplasm of MCF-7 and MDA-MB-231 cells, and mainly distributed around the nucleus. Green fluorescence was observed in almost all cells (Fig. 3a), indicating that the serum EVs of BC patients could be absorbed by BC cells.

EVs (0, 20, 40, 60 µg/mL) were added into MCF-7 and MDA-MB-231 cells. When culturing at 24, 48 and 72 h, MTT assay showed that the serum EVs from BC patients significantly stimulated the proliferation of MCF-7 and MDA-MB-231 cells, but the serum EVs from healthy subjects and benign individuals could not promote proliferation of MCF-7 and MDA-MB-231 cells. The promoting effect on proliferation was related to the concentration of EVs. When the concentration of EVs was 0–40 µg/mL, the higher the concentration, the stronger the promoting effect; when the concentration exceeded 40 µg/mL, the promoting effect of EVs reached a plateau (Fig. 3b–d). Therefore, 40 µg/mL of EVs and co-culture for 48 h were selected for the following experiments. In order to study whether the substances carried by EVs affect the proliferation of BC cells, we used SDS to treat the serum EVs (40 µg/mL) from BC patients and destroy the membrane structure of EVs. The results showed that after destroying the membrane structure, the promotion effect of EVs on the proliferation of BC cells disappeared (Fig. 3b), which indicated that the substances carried by EVs played a role in promoting the proliferation of BC cells. Then we tested the NEAT1 expression in MCF-7 cells before and after the treatment of serum EVs by RT-qPCR, and found that the expression difference of NEAT1 in MCF-7 cells after the treatment of serum EVs from BC patients was the most obvious (Fig. 3e).

To find out effects of overexpression of NEAT1 in EVs on BC cell invasion and migration, we conducted Transwell assay and scratch test. As shown in Fig. 3f–k, the serum EVs from BC patients with high NEAT1 expression significantly promoted invasion and migration of MCF-7 and MDA-MB-231 cells (*p* < 0.05). But the serum EVs from healthy subjects and benign individuals could not promote the invasion and migration of MCF-7 and MDA-MB-231 cells (all *p* > 0.05).

In addition, the lung metastasis model of BC in nude mice was established by injection of highly invasive MDA-MB-231 cells to verify the effect of EVs overexpressing NEAT1 on BC metastasis in vivo. The nude mice were sacrificed and the lung tissues were removed 45 days after the establishment of lung metastasis model. HE staining showed that compared with nude mice injected with PBS, the size and number of lung metastases were increased significantly in nude mice with high NEAT1 expression in serum EVs from BC patients (all *p* < 0.05). But the serum EVs from healthy subjects and benign individuals did not influence the size and number of lung metastases (all *p* > 0.05) (Fig. 3l).

### High expression of NEAT1 in EVs promotes chemotherapy resistance in BC cells

**Fig. 4:**
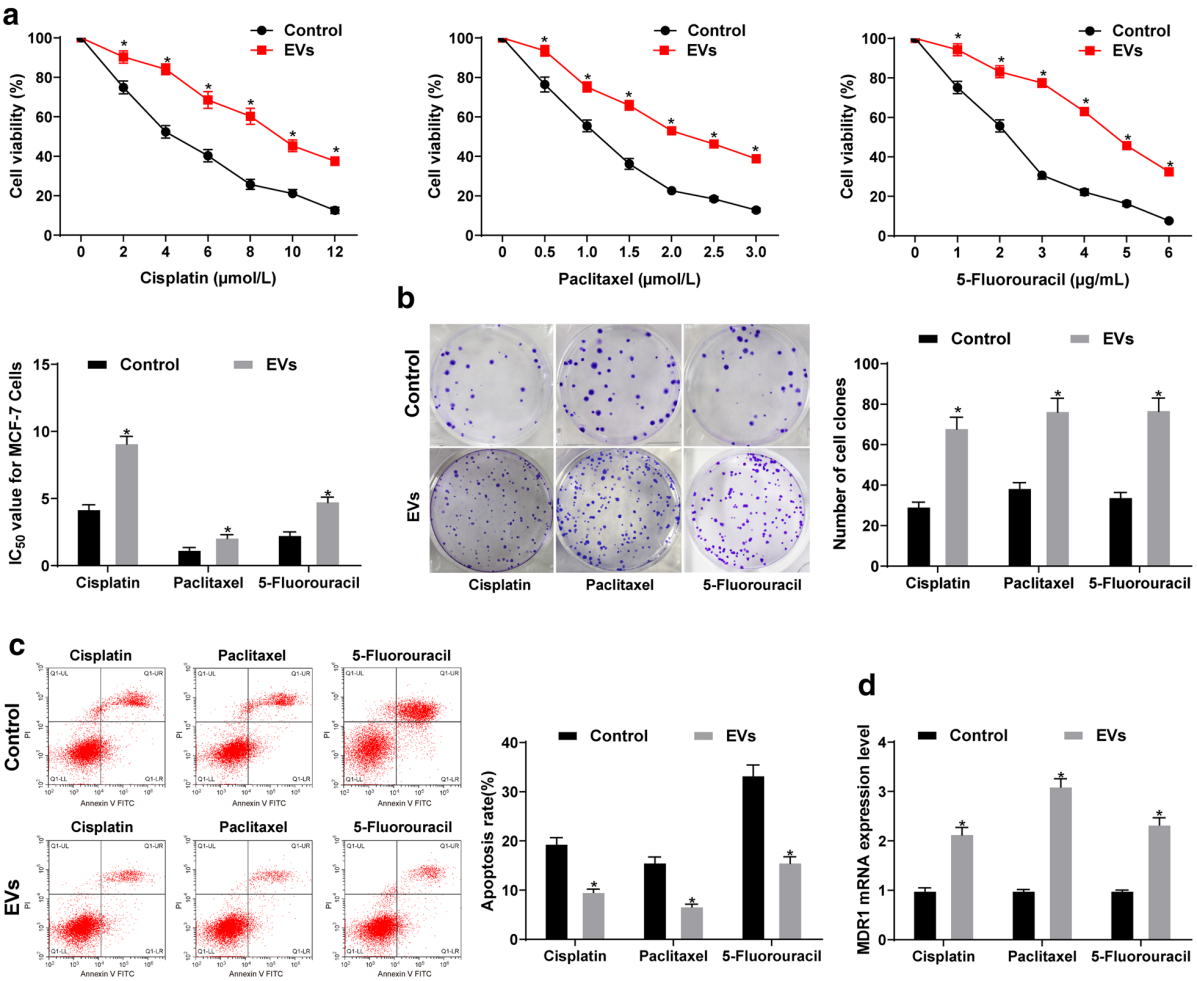
High NEAT1 expression in EVs promotes chemotherapy resistance in BC cells. **a** MTT assay was applied to measure MCF-7 cell proliferation; Cisplatin, paclitaxel and 5-fluorouracil were used to treat the cells respectively. The cell activity decreased with the increase of drug concentration. EVs weakened the drug effect and increased cell activity; **b** colony formation assay was utilized to detect MCF-7 cell colony formation ability; EVs increased the colony formation of MCF-7 cells under the action of drugs; **c** flow cytometry was utilized to determine MCF-7 cell apoptosis; EVs decreased the MCF-7 cell apoptosis under the action of drugs; **d** RT-qPCR was applied to detect MDR1 expression in MCF-7 cells; EVs increased MDR1 expression in MCF-7 cells under the action of drugs. Three independent experiments were performed; data were analyzed using two-way ANOVA, and Tukey’s multiple comparisons test was used for pairwise comparisons after ANOVA; * compared with the control group, *p* < 0.05

From above analyses, we confirmed that the EVs from BC patients promoted the proliferation, invasion and migration of BC cells, and we attempted to verify whether the EVs from BC patients affect the cell resistance. The effects of different concentrations of 5-fluorouracil, cisplatin and paclitaxel on the proliferation of MCF-7 cells treated with EVs were checked by MTT assay, and IC_50_ was calculated. The sensitivity of EVs-treated cells to cisplatin, paclitaxel and 5-fluorouracil was lower, while cisplatin IC_50_, paclitaxel IC_50_ and 5-fluorouracil IC_50_ were significantly higher than those of normal cells treated with PBS (all *p* < 0.05) (Fig. 4a).

The colony formation assay revealed that the inhibition of cisplatin, paclitaxel and 5-fluorouracil on the colony formation of MCF-7 cells was significantly reduced after EV treatment (all *p* < 0.05) (Fig. 4b).

The results of flow cytometry displayed that compared to cells treated with PBS, EVs-treated cells showed significantly inhibited apoptosis induced by cisplatin, paclitaxel and 5-fluorouracil (all *p* < 0.05) (Fig. 4c).

MDR1 expression was detected by RT-qPCR to explain the drug resistance of cells. MDR1 mRNA expression in EVs-treated cells was obviously higher than that in PBS-treated cells (all *p* < 0.05) (Fig. 4d). These results suggested that EVs from BC patients significantly enhanced the resistance of BC cells to chemotherapeutic drugs.

### NEAT1 silencing inhibits the promotion of BC cell invasion and migration induced by EVs

**Fig. 5:**
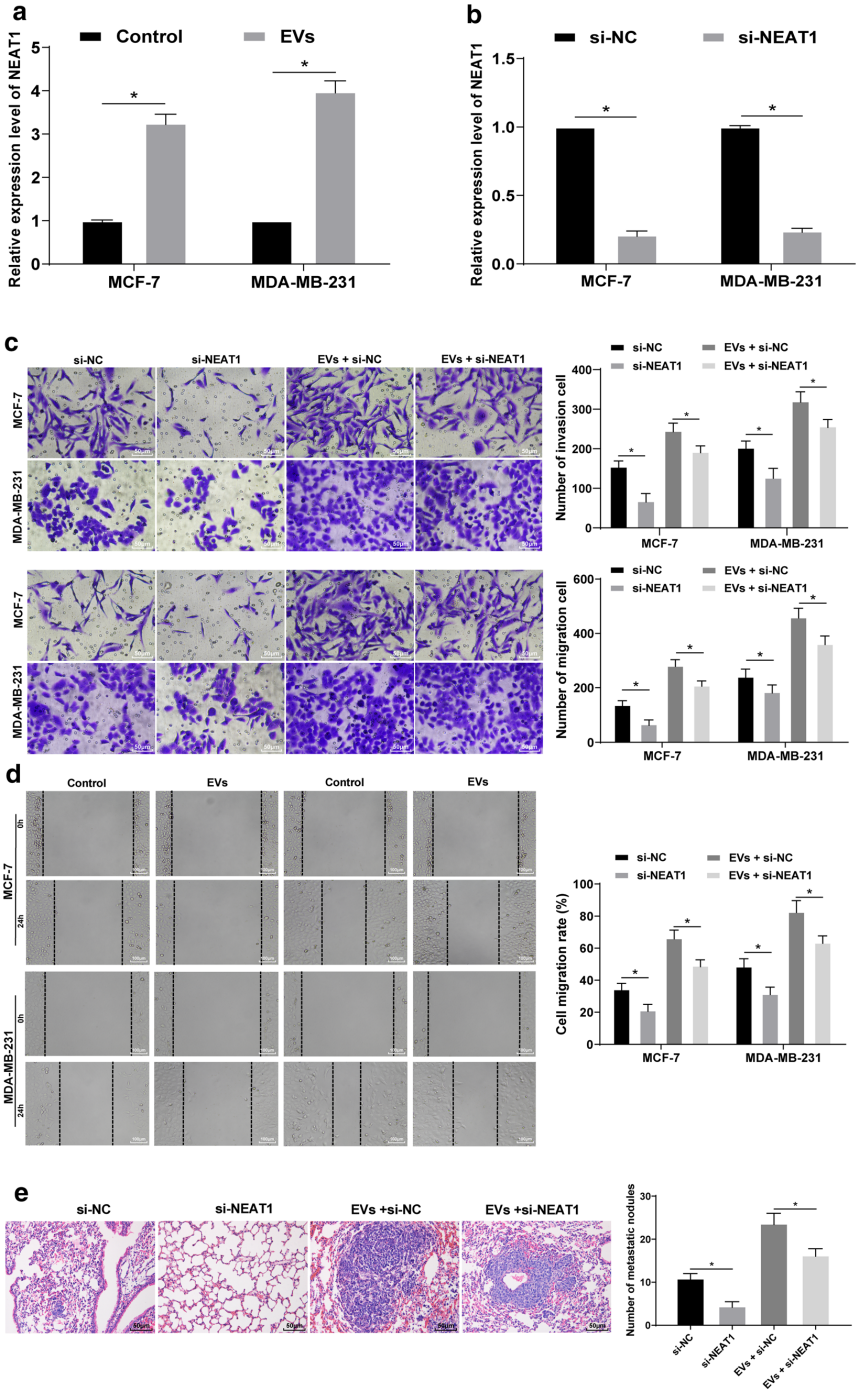
NEAT1 silencing inhibits EVs-induced promotion of BC cell invasion and migration. **a** RT-qPCR was applied to detect NEAT1 expression after EV treatment; NEAT1 expression in MCF-7 and MDA-MB-231 cells after treatment of serum EVs from BC patients was upregulated; **b** RT-qPCR was applied to detect NEAT1 expression in MCF-7 and MDA-MB-231 cells after transfection of NEAT1 siRNA; **c** transwell assay was utilized to measure MCF-7 and MDA-MB-231 cell invasion; **d** scratch test was applied to measure MCF-7 and MDA-MB-231 cell migration; **e** lung metastases in nude mice were applied to detect metastases in vivo. Three independent experiments were performed; * *p* < 0.05; data in panels **a**–**h** were analyzed using two-way ANOVA, and data in panel **i** were analyzed using one-way ANOVA. Tukey’s multiple comparisons test was used for pairwise comparisons after ANOVA

Through the above experiments, we knew that highly expressed NEAT1 in EVs could promote invasion and metastasis, as well as chemotherapy resistance in BC cells. NEAT1 expression in MCF-7 and MDA-MB-231 cells was increased significantly after EVs treatment (*p* < 0.05) (Fig. 5a). Therefore, we suggested that BC cell-derived EVs may enhance NEAT1 expression in BC cells by releasing NEAT1, thus promoting invasion and metastasis of BC cells and chemotherapy resistance.

NEAT1 siRNA plasmid was transfected into MCF-7 and MDA-MB-231 cells. After transfection for 24 h, NEAT1 expression in MCF-7 and MDA-MB-231 cells was decreased notably (all *p* < 0.05) (Fig. 5b), and migration and invasion of MCF-7 and MDA-MB-231 cells were inhibited markedly (Fig. 5c–h). The invasion and migration abilities of MCF-7 and MDA-MB-231 cells treated with interfering NEAT1 plus EVs were noticeably lower than those of cells treated with EVs alone (all *p* < 0.05) (Fig. 5c, d). In vivo experiments implied that cells treated with EVs + si-NC and cells treated with EVs + si-NEAT1 had obvious metastasis, but the size and number of lung metastases in cells treated with EVs + si-NEAT1 were significantly smaller than those in cells treated with EVs + si-NC (all *p* < 0.05) (Fig. 5e). These results suggested that inhibition of NEAT1 expression could reverse effects of EVs on BC cell invasion and migration.

### NEAT1 silencing inhibits EVs-induced promotion of chemotherapy resistance of BC cells

**Fig. 6:**
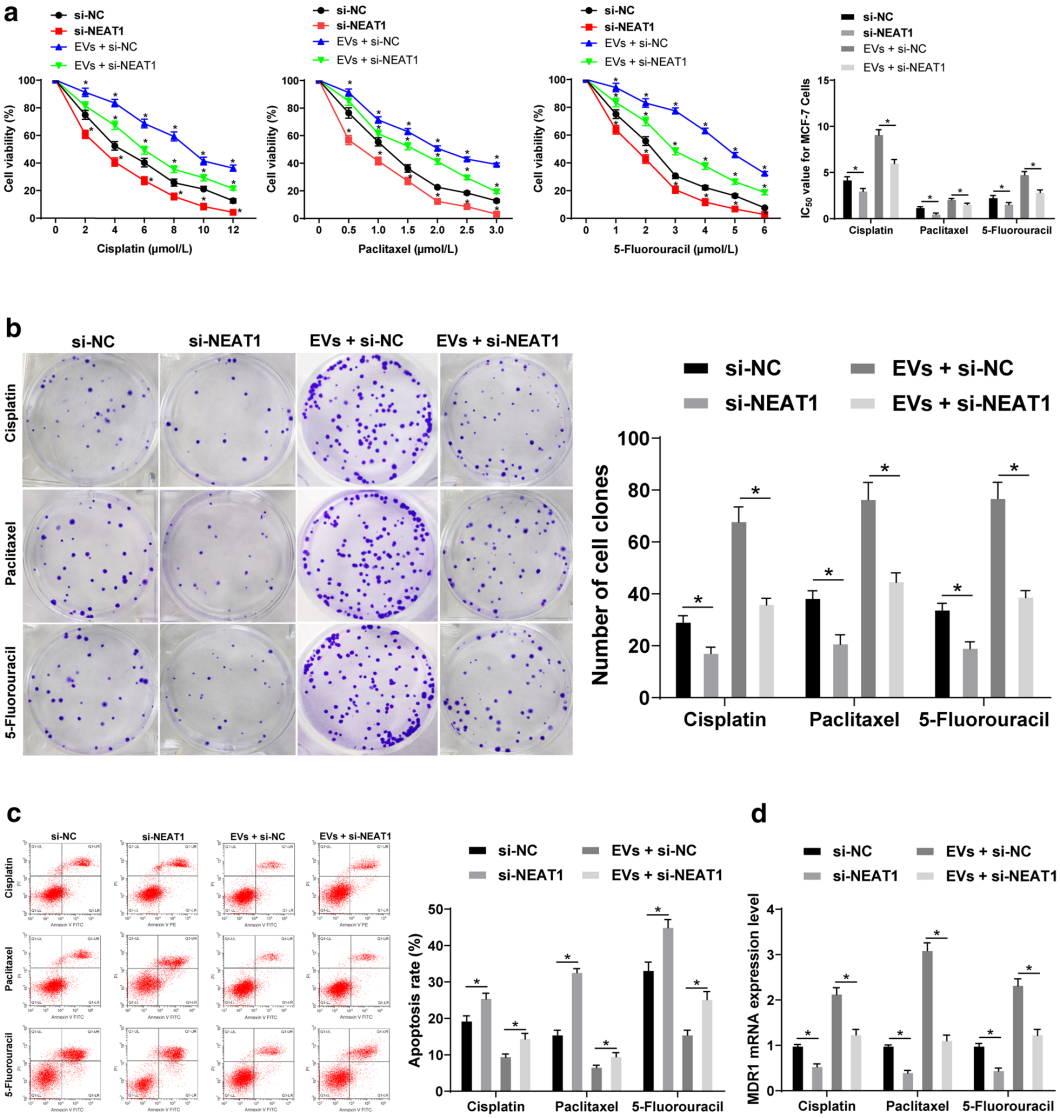
NEAT1 silencing inhibits EVs-induced promotion of chemotherapy resistance of BC cells. **a** MTT assay was applied to check the proliferative inhibition of MCF-7 cells; si-NEAT1 treatment decreased cell viability and si-NEAT1 + EVs increased cell viability; **b** colony formation assay was utilized to measure colony formation ability of MCF-7 cells; **c** flow cytometry was applied for detection of MCF-7 cell apoptosis; **d** RT-qPCR was utilized to detect MDR1 mRNA expression in MCF-7 cells. Three independent experiments were performed; * *p* < 0.05; data were analyzed using two-way ANOVA, and Tukey’s multiple comparisons test was used for pairwise comparisons after ANOVA

Different concentrations of cisplatin, paclitaxel and 5-fluorouracil were used to treat MCF-7 cells with low expression of NEAT1 and EVs. MTT assay showed that the proliferative inhibition rate of cells treated with EVs and si-NEAT1 was remarkably higher than that of cells treated with EVs and si-NC (all *p* < 0.05) (Fig. 6a). The inhibitory effect of each chemotherapeutic drug on colony formation ability and apoptotic induction effect in cells treated with EVs and si-NEAT1 were stronger than those in cells treated with EVs and si-NC, and MDR1 expression was lower than that in cells treated with EVs and si-NC (all *p* < 0.05) (Fig. 6b**–**d). These results suggested that silencing NEAT1 could inhibit EVs-induced chemotherapeutic resistance in BC, and EVs could increase NEAT1 expression in BC cells by releasing NEAT1 to BC cells, thus promoting BC cell invasion, metastasis and chemotherapeutic resistance.

### NEAT1 sponges miR-141-3p to regulate KLF12 expression

**Fig. 7:**
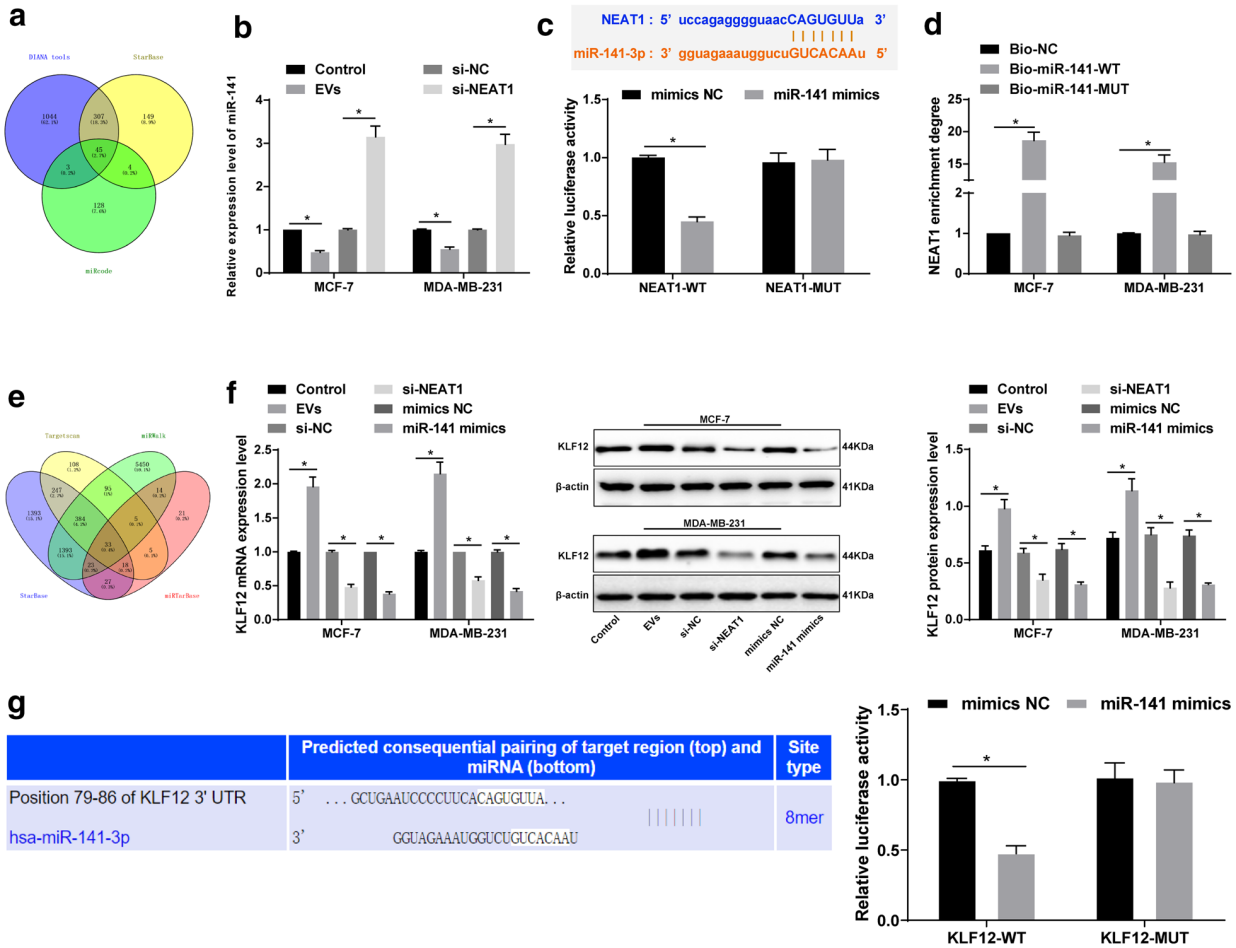
NEAT1 sponges miR-141-3p and KLF12 is the direct target gene of miR-141-3p. **a** Venn map of predicting miRs downstream of NEAT1 from DIANA tools (blue), miRcode (green) and StarBase (yellow) databases respectively; **b** RT-qPCR was applied to detect miR-141-3p expression in cells; **c** Bioinformatics website (http://starbase.sysu.edu.cn/) [45] and dual-luciferase reporter gene assay were applied to confirm the relationship between miR-141-3p and NEAT1; **d** RNA-pull down assay was used to verify the binding relationship between NEAT1 and miR-141-3p; **e** Venn map of predicting downstream genes of miR-141-3p from StarBase, Targetscan, miRWalk and miRTarBase databases; **f** RT-qPCR and Western blot analysis were utilized to detect levels of miR-141-3p and KLF12 in cells after overexpression of miR-141. **g** Bioinformatics website and dual-luciferase reporter gene assay were applied to confirm the targeting relationship between miR-141-3p and KLF12. Three independent experiments were performed; * *p* < 0.05; data were analyzed using two-way ANOVA, and Tukey’s multiple comparisons test was used for pairwise comparisons after ANOVA

In order to further understand the downstream mechanism of NEAT1, we used DIANA tools (http://carolina.imis.athena-innovation.gr/diana_tools/web/index.php?r=site%2Findex), miRcode (http://www.mircode.org/index.php) and Starbase (http://starbase.sysu.edu.cn/index.php) were used to predict the miRs downstream of NEAT1 and the results in three databases were intersected (Fig. 7a). Among them, we focused on miR-141-3p. The deletion of miR-141 promoted the dedifferentiation of BC cells [17]. We speculated that NEAT1 promotes invasion, metastasis and chemotherapy resistance of BC cells by binding to miR-141-3p. miR-141-3p expression in MCF-7 and MDA-MB-231 cells decreased significantly after EV treatment, increased significantly after interfering NEAT1 expression (all *p* < 0.05) (Fig. 7b). The on-line prediction software and dual-luciferase reporter gene assay found a targeting binding relationship between NEAT1 and miR-141-3p (Fig. 7c). Furthermore, RNA-pull down assay showed that Bio-miR-141-WT could promote the enrichment of NEAT1 around it (*p* < 0.05), but Bio-miR-141-MUT could not. It was confirmed that NEAT1 could reduce the degree of miR-141-3p dissociation in BC cells by binding to miR-141-3p (Fig. 7d).

Similarly, we use the StarBase, Targetscan (http://www.targetscan.org/vert_71/), miRWalk (http://mirwalk.umm.uni-heidelberg.de/), miRTarBase (http://mirtarbase.mbc.nctu.edu.tw/php/index.php) to screen out the downstream target genes of miR-141-3p and the intersection was taken (Fig. 7e). Then through literature search, we found that KLF12 is involved in tumor invasion and apoptosis (PMID: 27278159), and there are little studies of KLF12 in NC. The mRNA and protein levels of KLF12 in MCF-7 and MDA-MB-231 cells were increased significantly after EV treatment, but decreased after transfection with NEAT1 siRNA (all *p* < 0.05). After transfecting miR-141-3p mimic into MCF-7 and MDA-MB-231 cells, miR-141-3p expression increased, while KLF12 levels were decreased significantly (all *p* < 0.05) (Fig. 7f). In addition, bioinformatics prediction and dual-luciferase assay confirmed that KLF12 was the direct target gene of miR-141-3p (Fig. 7g). Therefore, we confirmed that NEAT1 released by BC cell-derived EVs could competently bind to miR-141-3p and upregulate KLF12 expression, thereby promoting the invasion, metastasis and chemotherapy resistance of BC.

## Discussion

Although neoadjuvant and systemic chemotherapies have been widely used for BC treatment, successful molecular-targeted therapies of any extent are still unavailable nowadays [23]. BC patients usually die of cancer recurrence even after adjuvant therapies and radical surgery [24]. NEAT1 accelerates BC cell invasion and proliferation through the regulation of miR-218 [25]. In this study, we explored the effects of NEAT1 in EVs on invasion and migration in BC cells through miR-141-3p and KLF12. Collectively, the data revealed that NEAT1 released by EVs originated from BC cells could bind to miR-141-3p and upregulate KLF12 expression, thereby promoting BC cell invasion, migration and chemotherapy resistance.

Tumor-derived EVs transfer miRs and lncRNAs in recipient cells to modulate the microenvironment and facilitate cancer metastasis [26]. NEAT1 expression in serum EVs from BC patients was higher than that of patients with benign individuals and healthy controls. NEAT1 is overexpressed in luminal A, luminal B, HER2^+^, and basal-like tumors [27], and high NEAT1 expression is related to poor prognosis and short overall survival in human cancers [28]. A recent study further indicated that higher NEAT1 expression has positively association with lymph node metastasis and tumor node metastasis staging in BC patients [25], which was in line with our results. Additionally, BC patients with high expression of NEAT1 in serum EVs had higher lymph node metastasis rate, PR, ER and Ki-67 positive rates. ER and PR are the primary predictive molecular markers for BC, and Ki-67 is a proliferation marker related to BC invasion and recurrence [29].

Cancer-derived EVs are important in tumor metastasis and chemoresistance by transmission of lncRNAs [13, 14]. High expression of NEAT1 in serum EVs facilitated higher lymph node metastasis rate, and promoted BC cell invasion, migration, chemotherapy resistance, and in vivo metastasis. EV levels are higher in the serum of BC patients compared to normal subjects and play roles in the context of tumor growth and the stimulation of metastasis by delivering cargos [30]. EVs originated from BC cells facilitate tube formation and migration of endothelial cells to accelerate angiogenesis, inducing cancer metastasis [31], and stimulate tumor invasion and metastasis, tumor growth as well as angiogenesis [32]. Similarly, NEAT1 upregulation in hypoxia contributes to increased cellular proliferation and tumorigenesis [33], and hypoxia condition in BC is a major trigger for exosome secretion [34]. Besides, chemotherapy is important against BC, but its development is mainly restricted by drug resistance [35]. Li et al. [36] proposed that NEAT1 plays a pivotal role in 5-fluorouracil resistance in BC. A former in vitro study, docetaxel resistance could be acquired by delivery of P-glycoproteinvia exosomes in MCF-7 BC cells [37]. Drug-resistant BC cells function on the release of EVs, and the blockade of EV transfer from drug-resistant BC cells to sensitive BC cells might improve treatment outcomes [38]. In addition, NEAT1 silencing inhibited the promotion of invasion, metastasis, and chemotherapy resistance of BC cells induced by EVs. NEAT1 downregulation inhibits BC cell metastasis and invasion by reversing the epithelial-mesenchymal transition phenotype [36]. NEAT1 is upregulated in ovarian cancer and its knockdown promotes paclitaxel sensitivity of ovarian cancer [39]. However, there is little research on the relevance of tumor-derived exosomes and NEAT1 in BC development. This highlights the innovation of our study to a great degree.

Furthermore, bioinformatics prediction, dual-luciferase assay and RNA-pull down assay acclaimed that EV-released NEAT1 sponged miR-141-3p and KLF12 was the direct target gene of miR-141-3p. The competing endogenous RNA (ceRNA) network of lncRNA-miR-mRNA always exists in tumor modulation. The tumor driver NEAT1 acts as a ceRNA to regulate the miR-34b-5p-GLI1 axis, further affecting the proliferation of diffuse large B-cell lymphoma [40]. Low expression of miR-141 in serum of BC patients is related to poor disease-free survival rate and overall survival rate [41]. miR-141 overexpression grants chemoresistance in docetaxel-sensitive MCF-7 and MDA-MB-231 cells [42]. KLF12 is at high levels in in basal-like breast carcinoma [19]. KLF12 affects the drug resistance of osteosarcoma cells [43]. Besides, miR-141 can increase resistance against detachment-induced cell death by inhibiting KLF12 expression in ovarian cancer [44]. That’s to say, the promoting effects of high NEAT1 expression in EVs on BC cell invasion, migration and chemotherapy resistance were achieved by sponging miR-141-3p and upregulating KLF12.

To summarize, our study provided strong evidence that NEAT1 released by EVs derived from BC cells could competently bind to miR-141-3p and upregulate KLF12 expression, thereby promoting BC cell invasion, metastasis and chemotherapy resistance. These results are promising in prospect of promoting future treatment of BC, thereby providing references for optimizing individual treatment for BC patients. Though our findings offer therapeutic implication in treatment for BC, the experimental results and effective translation into clinic practice still need future validation. Additionally, we may conduct further research in our later research to determine the related mechanism of NEAT1-regulated downstream factors in BC cell resistance to different drugs.

## Data Availability

All the data generated or analyzed during this study are included in this published article.
